# Advancements in prospective single-cell lineage barcoding and their applications in research

**DOI:** 10.1101/gr.278944.124

**Published:** 2024-12

**Authors:** Xiaoli Zhang, Yirui Huang, Yajing Yang, Qi-En Wang, Lang Li

**Affiliations:** 1 College of Nursing, University of South Florida, Tampa, Florida 33620, USA;; 2 College of Pharmacy, The Ohio State University, Columbus, Ohio 43210, USA;; 3 Department of Radiation Oncology, Comprehensive Cancer Center, The Ohio State University, Columbus, Ohio 43210, USA;; 4 Department of Biomedical Informatics, College of Medicine, The Ohio State University, Columbus, Ohio 43210, USA

## Abstract

Single-cell lineage tracing (scLT) has emerged as a powerful tool, providing unparalleled resolution to investigate cellular dynamics, fate determination, and the underlying molecular mechanisms. This review thoroughly examines the latest prospective lineage DNA barcode tracing technologies. It further highlights pivotal studies that leverage single-cell lentiviral integration barcoding technology to unravel the dynamic nature of cell lineages in both developmental biology and cancer research. Additionally, the review navigates through critical considerations for successful experimental design in lineage tracing and addresses challenges inherent in this field, including technical limitations, complexities in data analysis, and the imperative for standardization. It also outlines current gaps in knowledge and suggests future research directions, contributing to the ongoing advancement of scLT studies.

As the basic structural and functional units of life, deciphering the lineage and developmental trajectories of individual cells has been a crucial pursuit in understanding the process of organ and tissue formation, as well as the progression of diseases. A profound exploration into the molecular mechanisms dictating cell differentiation, organization, fate, and function has long been the focus point within the fields of developmental biology and pathological processes (Wagner and Klein 2020).

In the context of diseases, particularly in conditions like cancer, it is imperative to elucidate the origin of the disease process, identifying, and isolating the rare subset of cells resilient to treatment, thereby giving rise to therapeutic resistance. This endeavor is critical in developing innovative preventive and therapeutic strategies in disease treatment and management. Conventional cell identification heavily relies on specific cell surface biomarkers or their combinations. However, the scarcity of comprehensive biomarkers and the nonspecific nature of many biomarkers pose challenges. Cells identified through this method frequently comprise a heterogeneous mix, introducing uncertainty and ambiguity. This limitation not only increases the risk of missing the identification of cells of interest but also introduces the potential of misidentifying the correct cell groups. These inaccuracies introduce biases into the depiction of the complexity of biological processes, impeding an accurate understanding of the molecular mechanisms involved (Kester and van Oudenaarden 2018).

The recent breakthrough in single-cell RNA sequencing (scRNA-seq) has revolutionized our ability to profile tens of thousands of individual cells across various differentiation stages concurrently. This technological advancement provides unparalleled resolution, unveiling novel cell types, and shedding light on previously undiscovered mechanisms (Grun et al. 2015; Zeisel et al. 2015; Cannoodt et al. 2016; Kester and van Oudenaarden 2018). Computational algorithms like Monocle and RNA velocity have emerged to predict cell lineage differentiation trajectories based on transcriptomic similarity and pseudo-temporal ordering (La Manno et al. 2018; Cao et al. 2019). However, while scRNA-seq data offer insights into the transcriptomic landscape, it cannot establish direct long-term dynamic relationships between cells and their progeny or among different individual cells (Wagner and Klein 2020). In addition, trajectory descriptions derived from pseudotime methods may not represent the true lineage differentiation path of a progenitor population without ground truth evidence support. In recent years, the incorporation of inheritable cell-specific DNA barcodes in lineage tracing, followed by barcode sequencing, has emerged as a powerful approach. This technique allows the prospective tracking of millions of individual cells simultaneously, providing a unique opportunity to trace cellular lineages over time (Wagner and Klein 2020). The integration of single-cell lineage tracing (scLT) and single-cell transcriptomics presents a significant opportunity to explore clonal complexity. This integration allows for the connection of cells from the present to their historical lineage. Additionally, the refinement of clonal dynamics is achieved by leveraging transcriptome-derived differentiation trajectories and assessing gene expression changes over time (Kester and van Oudenaarden 2018). In contrast to intentionally introducing heritable tracers (DNA barcodes) for prospective lineage tracing, naturally occurring somatic mutations that accumulate throughout an organism's lifetime have been used for retrospective lineage tracing to study development, especially with the advancement of sequencing technologies (Dou et al. 2018; Wu et al. 2019). However, the relative infrequency of somatic mutation produces phylogenies of limited resolution (VanHorn and Morris 2021).

As a cutting-edge technology, the current scLT method seamlessly integrates scLT with transcriptomics, enabling simultaneous detection of cell state transition, clonal relationship, and elucidation of the molecular mechanisms in cell fate determination (Chen et al. 2022). While there has been extensive discussions on the fundamental concepts, computational tools, and applications of scLT (Kester and van Oudenaarden 2018; McKenna and Gagnon 2019; Wagner and Klein 2020; VanHorn and Morris 2021; Chen et al. 2022), there remains a notable gap in detailed discussions regarding the technical considerations and caveats inherent in performing scLT experiments. In this review, we will first explore the current landscape of prospective lineage tracing technologies featuring inheritable genetic features. Next, we will highlight their applications and power across various biological research fields, with a specific emphasis on scLT utilizing viral integration DNA barcodes. Following this, we will discuss the experimental details and challenges encountered in practical applications. Finally, we will briefly speculate the future directions that this evolving field may take, providing insights into the potential avenues of exploration.

## Lineage tracing—the past and the present

**Figure 1.**
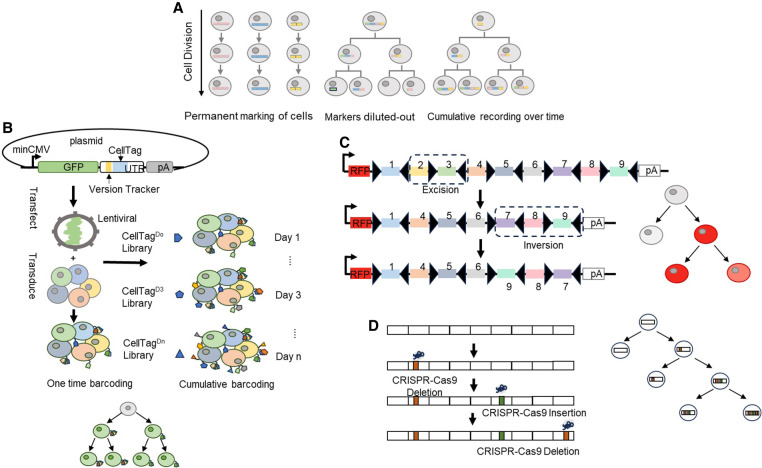
Prospective DNA barcoding strategies. (*A*) Conceptual illustration of lineage tracing: Cells can be permanently labeled and inherited by offspring cells, markers on labeled cells become increasingly diluted with subsequent divisions, or cells can be cumulatively labeled and recorded over time. (*B*) Lentivirus integration barcoding: The DNA barcode sequence is inserted into the 3′ UTR of the GFP reporter gene and packaged into a lentiviral vector. Cells of interest are transduced and labeled with a unique DNA sequence through either one-time labeling (*left*) or continuous labeling over time (*right*). (*C*) Cre-recombinase-based barcoding: Cre recombinase recognizes the LoxP site on the introduced gene, then recombination results in inversion or deletion of the DNA sequence of the LoxP site depending on the orientation of these sites. (*D*) CRISPR–Cas9-based barcoding: During cell differentiation, Cas9 induces double-strand breaks, and incomplete nonhomologous end joining (NHEJ) repair leads to the generation of barcode insertions and deletions over time, serving as a valuable tool for lineage tracing.

Lineage tracing serves as the gold standard in developmental biology, allowing for the inference of relationships between progenitors and their offspring. Figure 1A illustrates the overall concept of lineage tracing, which can be either permanent, diluted out, or accumulative in tracking cells over time. This technique involves tracking the descendants of single cells to define the developmental trajectory of cell lineages (VanHorn and Morris 2021; Chen et al. 2022). It was initially performed to track cells over time through visualization (Deppe et al. 1978) by utilizing different strategies including creating chimeric embryos (Mintz 1967), engrafting cells from one species to another (Le Douarin and Teillet 1973), injecting vital dyes into a single founder cell of transparent organisms like *Caenorhabditis elegans* and zebrafish (Lawson et al. 1986; Pedersen et al. 1986; Stern and Fraser 2001), or later through the introduction of reporter genes (Turner and Cepko 1987; Frank and Sanes 1991). With the development of fluorescence-activated cell sorting (FACS) and corresponding single-cell isolation and cell transplantation technique, the introduction of reporter transgenes such as β-galactosidase or green fluorescence protein (GFP) into cells has become a powerful tool to assess cell proliferation and differential potential (Osawa et al. 1996; Quintana et al. 2008). Through virus transfection, transgenes integrate into the host genome; therefore, the descendants of these cells will inherit the transgenes and express a fluorescent protein, which can be easily visualized by microscopy. In this way, the offspring of the parental cells are labeled and traced allowing fate determination of their progenies (Kester and van Oudenaarden 2018). However, the expression of the fluorescence protein is normally controlled by site-specific recombinases such as Cre under the control of cell-type-specific promotors; therefore, its expression is confined into a group of cells instead of a single cell (Chen et al. 2022). This strategy often leads to sparsely distributed clones across a sample, making it challenging to distinguish clones from one another (Kester and van Oudenaarden 2018).

In response to the limitation of single fluorescence protein labeling, multicolor labeling methods such as Brainbow (Livet et al. 2007) and Confetti (Snippert et al. 2010) were developed. These methods introduce multiple fluorescent proteins to differentiate cells with various color combinations. However, despite the advancements provided by multicolor labeling, there are still challenges associated with this approach. The limited number of available fluorescent colors constrains the number of cells that can be confidently tracked. This limitation becomes particularly pronounced when dealing with the complex combinations of dose and labeling time during the initial testing phase. Moreover, the multicolor labeling method faces difficulty in distinguishing between primary cells and their progenies. This challenge can complicate the accurate tracking and interpretation of cell lineages over time. Despite these challenges, the development of multicolor labeling techniques has significantly improved the ability to trace cell lineages, offering valuable insights into the dynamics of cellular populations (Liu et al. 2020; Chen et al. 2022).

The emergence of DNA lineage barcoding technology represents a paradigm shift in cell lineage tracing. This method utilizes unique DNA sequences to prospectively label individual cells by inserting barcodes into the genome of host cells. These barcodes are inherited by offspring cells through cell division, allowing for precise lineage tracking. The number of potential barcodes increases exponentially with the increased length and multiplicity of the random nucleotide sequence, providing a vast array of unique labels. When combined with current single-cell sequencing techniques, DNA lineage barcoding offers unlimited potential to study cell behavior and fate over space and time. This powerful combination allows researchers to track the dynamics and behavior of individual cells and their progeny comprehensively. The inheritable nature of this method enables the long-term tracking of cell lineages, offering a detailed view of developmental processes and responses to environmental cues (Wagner and Klein 2020; Chen et al. 2022). As a result, DNA lineage barcoding has been widely used in studies focused on cell differentiation and evolution (Naik et al. 2014; Biddy et al. 2018; Rodriguez-Fraticelli et al. 2018; Weinreb et al. 2020), cell heterogeneity and cell fate during drug resistance (Nguyen et al. 2014b; Bhang et al. 2015; Lan et al. 2017; Emert et al. 2021), tumor-initiating cells (Wang et al. 2021), and metastasis in cancer (Merino et al. 2019). Here, we describe the three major types of exogenous barcode delivery systems (Wagner and Klein 2020; Morgan et al. 2021; VanHorn and Morris 2021; Chen et al. 2022) that are used for DNA barcode lineage tracing as illustrated in Figure 1.

### Integration barcodes

This method uses lentivirus/retrovirus, transposon, or episome-based delivery system to integrate a short exogenous DNA sequence to the genomics of cells (Fig. 1B; Chen et al. 2022; Shlyakhtina et al. 2023). The DNA segment can be synthesized to include consecutive random sequences named as type I barcodes or random nucleotides interspersed with fixed nucleotides as type II barcodes (Lu et al. 2011; Levy et al. 2015; Kebschull et al. 2016; Rodriguez-Fraticelli et al. 2018; Bramlett et al. 2020). The synthetic DNA sequences are embedded within a viral construct or flank the integration sites of transposons that can be easily quantified by high-throughput sequencing (Bramlett et al. 2020). In addition, the DNA segments are typically inserted in the 3′ UTR after the coding region of fluorescent proteins, which allows for convenient FACS sorting to retrieve the barcoded cells. Each cell is tagged with a specific barcode sequence of a given length, such that the number of barcodes is equivalent to 4^*N*^, where *N* is the length of the DNA barcode sequence. The vast diversity of barcode sequences provides the potential to track millions of cells at the same time, making it possible to study complex cellular populations and dynamic processes (Wang et al. 2021). Integration barcodes are usually used as static (invariable) barcodes to label a pool of cells, allowing for revealing clonal potency such as self-renewal and multipotent properties directly by identifying cell types that share the same barcodes (Chen et al. 2022). It can also be used as cumulative barcodes with continuous delivery of barcodes during a developmental process to record the history of mitotic divisions as demonstrated in the somatic reprogramming study (Fig. 1B; Biddy et al. 2018). In this case, a multilayer clonal tree can be reconstructed, and the subclonal relationships from different cell types can be revealed by analyzing the clonal trees based on the number of barcodes (Chen et al. 2022).

Since its first publication (Lu et al. 2011), numerous studies have been performed to adapt or improve the integration barcoding system for lineage tracing in different organisms. Transposon integration has been applied to study the native fate of hematopoietic stem cells (HSCs) and multipotent progenitor cells using in vivo studies (Sun et al. 2014; Rodriguez-Fraticelli et al. 2018). Additionally, in the “TracerSeq” study (Wagner et al. 2018), it was employed in the reconstruction of single-cell lineage histories in zebrafish, leveraging gene expression landscapes. Recently, episomes were used for cell transfection and genomic integration in the Barcode decay Lineage Tracing-Seq (BdLT-Seq) study to investigate lineage-linked transcriptome plasticity (Shlyakhtina et al. 2023). Compared to other integration methods, the viral barcoding system, utilizing lentivirus for integration, has been used extensively in recent years (Eirew et al. 2015; Zeisel et al. 2015; Biddy et al. 2018; Kester and van Oudenaarden 2018; Cao et al. 2019; Guo et al. 2019; Merino et al. 2019; Bramlett et al. 2020; Weinreb et al. 2020; Emert et al. 2021; Ratz et al. 2022; Umeki et al. 2022; Wei et al. 2022).

The surge in high-throughput sequencing technology and reduced cost, particularly with the advent of scRNA-seq, aligns well with the high-throughput capabilities of viral DNA barcoding technology. Novel barcode libraries have been evolved to express DNA barcodes as RNA-transcripts that can be captured by scRNA-seq, such as LARRY (Weinreb et al. 2020), CellTagging (Biddy et al. 2018), Watermelon (Oren et al. 2021), BdLT (Shlyakhtina et al. 2023), ClonMapper (Gutierrez et al. 2021), Rewind (Emert et al. 2021), and others (Table 1). These methods typically involve the insertion of barcodes within the 3′ UTR of a fluorescence reporter gene. The expression of the fluorescence gene is governed by a constitutive promoter, ensuring consistent and predictable barcode capture. This design facilitates the simultaneous profiling of barcodes and single-cell transcriptomics through scRNA-seq, allowing for the labeling of individual cells and the construction of their fates at a single-cell resolution (VanHorn and Morris 2021). This synergy enables the simultaneous comparison of numerous individual cells, providing a direct and comprehensive assessment of cellular heterogeneity. This feature shows great advantage in ex vivo labeling, commonly applied to label millions of HSCs and cancer cells at the initiation of lineage tracing. Subsequently, their clonal dynamics are evaluated using scRNA-seq, demonstrating the potential for thorough and parallel exploration of cellular behavior (Chen et al. 2022). However, its applicability is constrained when it comes to in vivo labeling of tissues, organs, or organisms. This limitation arises from the difficulty in selecting appropriate time windows, tissue dissociations, and the challenge of controlling the number of barcodes per cell and the number of cells to be labeled (Kebschull and Zador 2018).

**Table 1.** Summary of prospective single-cell lineage tracing studies using viral DNA barcoding

Study	Editing system	Method	Read out	Barcode type	MOI	In vitro or in vivo (species)	Sequencing	Reference
Zebrafish embryo lineage development	Transposon	TracerSeq	scRNA-seq	GFP + 20 bp	∼54% of cells with barcode	Zebrafish	InDrops Illumina	Wagner et al. (2018)
Lineage-linked cancer transcriptome plasticity	Episome	BdLT-Seq	scRNA-seq	GFP + 12 bp	0.05	In vitro	10 × 3′	Shlyakhtina et al. (2023)
Mouse embryonic fibroblast (MEF) reprogramming	Lentivirus	CellTag	scRNA-seq	GFP + 8 bp	3–4	In vitro	Drop-seq, 10 × 3′	Biddy et al. (2018)
Multiple samples with species mixing for sample multiplexing	Lentivirus	CellTag	scRNA-seq snRNA-seq	GFP + 8 bp	0.5% of cells with barcode	In vitro and mouse	10 × 3′	Guo et al. (2019)
Nature protocol from Morris	Lentivirus	CellTag	scRNA-seq	GFP + 8 bp	3–4	In vitro	10 × 3′	Kong et al. (2020)
Fate-specific gene regulatory changes in MEF to endoderm transition	Lentivirus	CellTag-multi	scRNA-seq/scATAC-seq	GFP + read 1N + 28 bp + read 2N + RT priming site	∼2.25	In vitro	10x multiome	Jindal et al. (2023)
Mouse hematopoietic differentiation	Lentivirus	LARRY	scRNA-seq	GFP + 28 bp	98% of cells with GFP signal	In vitro and mouse	InDrops Illumina	Weinreb et al. (2020)
Mouse brain developmental neurogenesis	Lentivirus	TREX/Space-TREX	scRNA-seq/ST^a^	EGFP + 30 bp	NA	Mouse	10 × 3′	Ratz et al. (2022)
Stem cell hierarchies in rhabdomyosarcoma	Lentivirus	LARRY	scRNA-seq	GFP + 28 bp	0.3	In vitro	10 × 3′	Wei et al. (2022)
Clonal dynamics in tumor evolution and treatment (CLL)	Lentivirus	ClonMapper	scRNA-seq CROP-seq	sgRNA barcodes with BFP + 20 bp	0.1	In vitro	10 × 3′	Gutierrez et al. (2021)
Cell fate in melanoma drug resistance	Lentivirus	Rewind	RNA FISH	GFP + gDNA (100 bp)	<0.5	In vitro	Targeted DNA-seq	Emert et al. (2021)
Cycling persister cells in lung cancer drug resistance	Lentivirus	Watermelon	scRNA-seq live cell imaging	mNeonGFP + 90 bp (semirandom seq)	0.3	In vitro	10 × 3′	Oren et al. (2021)
Clonal diversity in TNBC primary and metastatic tumors	Lentivirus	Viral barcoding	scRNA-seq	GFP + 98 bp (semirandom seq)	0.1–0.2	Mouse	10 × 3′	Merino et al. (2019)
Malignant clonal fitness in AML	Lentivirus	SPLINTR	scRNA-seq	GFP, BFP or mCherry + 60 bp (semirandom seq)	0.02–0.1	In vitro and mouse	10 × 3′	Fennell et al. (2022)
Mathematical framework to predict cancer therapeutic resistance	Lentivirus	COLBERT	scRNA-seq	BFP + 20 bp gRNA	<0.1	In vitro	10 × 3′	Johnson et al. (2020)
Rates, routes and drivers of lung metastasis in xenografts	Lentivirus	Cas9-based recorder	scRNA-seq	BFP + 205 bp triple-gRNAs	∼0.5	Mouse	10 × 3′	Quinn et al. (2021)

^a^(ST) Spatial transcriptomics.

### Cre recombinase-based DNA barcodes

This method relies on the Polylox rearrangement with the Cre-LoxP recombination system to create barcodes. Cre recombinase recognizes the specific DNA sequence called LoxP site on the introduced gene, allowing the sequence to be manipulated through LoxP site excision or inversion upon recombination (Fig. 1C). In a recent study, a large-size synthetic gene (2.1 kb) with 10 LoxP sites was integrated into the mouse genome to study HSC differentiation (Pei et al. 2019). Recombination with the Cre recombinase resulted in random deletion, inversion, or translocation of the flox sites from the DNA sequence, generating cell-specific genetic labels. In combination with sequencing technology, these specific barcodes were identified to evaluate cell differentiation (Pei et al. 2017, 2019; Wang et al. 2021). Although this system is frequently implemented in model systems to study tissue/cell dynamics and tissue maintenance in a tissue and time-specific manner, the limitation of this system is due to the Cre-LoxP properties (Kester and van Oudenaarden 2018; Wang et al. 2021). First, the Cre-LoxP system is prone to excision than inversion, leading to the reduced size of the target array over time and reduced barcode diversity (Wang et al. 2021). Second, the target array is normally long and repetitive because of the low diversity of recombinase recognition sites. To achieve high barcode diversity, the target array needs to contain multiple fragments that require the barcode to be read by long sequencing technology (Wang et al. 2021). Third, the induction of Polylox rearrangement can only occur once in cells similar to the viral barcoding, which limits the construction of multilevel phylogenetic trees (Kester and van Oudenaarden 2018). Lastly, the reporter expression used to label cells may be silenced in specific cell types as seen in retrovirus labeling (Walsh and Cepko 1992), which can mask genuine lineage relationships (VanHorn and Morris 2021). Recently, a novel digital, image-readable lineage recoding system called intMEMOIR (integrase-editable memory by engineered mutagenesis with optical in situ readout) based on site-specific serine integrates was developed to allow for simultaneous analysis of single-cell clonal history, transcriptional state, and spatial organization in the same tissue (Li et al. 2023), which can significantly overcome the limitations presented by the traditional Cre-LoxP system.

### CRISPR–Cas9 editing-based barcodes

This method uses CRISPR–Cas9-directed genome editing technology. The binding of Cas9 nuclease to a targeted region often creates short random insertions or deletions, called indels (Jao et al. 2013). These DNA marks can be inherited by all the descendent cells as traceable elements, allowing for later lineage reconstruction (Fig. 1D). This principle was first confirmed by McKenna et al. (2016) in zebrafish through applying the genome editing of synthetic target arrays for lineage tracing (GESTALT) system. In this study, CRISPR–Cas9 and guide RNAs (gRNAs) were injected into one-cell embryo to allow scarring in the target sequence to study the lineage contribution of early embryonic cells to adult zebrafish organs (McKenna et al. 2016). Modified CRISPR–Cas9 systems with increased barcode diversity such as mSCRIBE (mammalian synthetic cell recorder integrating biological events) (Perli et al. 2016) and Homing CRISPR barcode (Kalhor et al. 2018) were developed later. Several recent studies have integrated the CRISPR–Cas9 barcoding system with scRNA-seq, such as ScarTrace (Junker et al. 2017; Alemany et al. 2018), LINNAEUS (lineage tracing by nuclease-activated editing of ubiquitous sequences) (Spanjaard et al. 2018), scGESTALT (Raj et al. 2018), and studies performed by Chan et al. (2019). More recently, the establishment of CARLIN (Bowling et al. 2020; Wang et al. 2021), a mouse cell line for CRISPR array repair lineage tracing, and its improved line DARLIN (Li et al. 2023) has significantly increased lineage-barcoding capacity and recovery efficiency in the single-cell assay, enabling simultaneous cell lineage tracing, single-cell transcriptomics, and/or genome-wide methylation profiling in complex in vivo mammalian systems. The study with DARLIN found that cellular clonal memory is associated with genome-wide DNA methylation rather than gene expression or chromatin accessibility (Li et al. 2023). Additionally, iTracer (He et al. 2022) and CREST/snapCREST (Xie et al. 2023) were developed to incorporate both single-cell transcriptomics and spatial transcriptomics in CRISPR–Cas9-based lineage tracing to study cerebral organoid development and mouse brain development, respectively. Beyond their prevalent applications in developmental biology, Cas9-induced scarring barcodes have also been applied to trace cell plasticity and routes of tumor evolution and metastasis (macsGESTALT and others) (Morgan et al. 2021; Quinn et al. 2021; Simeonov et al. 2021; Yang et al. 2022) and studying temporal events during development and tumorigenesis (NSC-seq) (Islam et al. 2023). Furthermore, computational methods such as LinTIMaT (Zafar et al. 2020), DCLEAR (Gong et al. 2022), Cassiopeia (Jones et al. 2020), Startle (Sashittal et al. 2023), LinRace (Pan et al. 2023), and ConvexML (Prillo et al. 2023) have been developed for tree inference from lineage barcodes generated with CRISPR-based editing technology.

The CRISPR–Cas9 system enables the labeling of various tissues and organs across various organisms, generating high diversity in vivo barcodes over time. However, its reliance on the NHEJ repairing model results in a higher occurrence of deletions than insertions. Consequently, this tendency leads to the gradual shortening of CRISPR barcodes over time. Therefore, the practical diversity of barcodes generated by this system tends to be significantly lower than what is theoretically anticipated (Wang et al. 2021). (For further recent references and overviews of CRISPR barcodes, see Woodworth et al. [2017], Baron and van Oudenaarden [2019], Wagner and Klein [2020], and Sommer et al. [2023].)

## Viral DNA barcoding in developmental biology and cancer research

Among the three major DNA barcoding methods, the viral barcoding technique has been widely employed in developmental biology to study cell differentiation and heterogeneity (Brewer et al. 2016; Nguyen et al. 2018; Rodriguez-Fraticelli et al. 2018; Wagner et al. 2018; Lu et al. 2019; Weinreb et al. 2020; Ratz et al. 2022). Furthermore, this method has gained significant attention for investigating cell behaviors in the context of cancer (Turner and Cepko 1987; Nguyen et al. 2014b, 2015; Bhang et al. 2015; Eirew et al. 2015; Lan et al. 2017; Woodworth et al. 2017; Merino et al. 2019; Bramlett et al. 2020; Gutierrez et al. 2021; Oren et al. 2021; Quinn et al. 2021; Umeki et al. 2022; Wei et al. 2022). Its applications include unveiling the cellular origins of cancer genesis, relapse, and metastasis, as well as exploring the heterogeneous responses of cells to drug treatment.

In these studies, a common methodology involves the ex vivo labeling of target cells, which can include cells derived from cell lines, patient samples, or animal models. The labeled cells are then expanded and divided, with one portion saved as the original control and other portions subsequently tracked either in in vitro cell culture or in animal models (Bramlett et al. 2020). The assessment of cell dynamics and behaviors is then conducted by establishing connections between present cells and their origins, utilizing results obtained from high-throughput sequencing technology.

The advent of scLT with integrative DNA barcodes has revolutionized the field, offering unprecedented insights into cellular dynamics and developmental processes (VanHorn and Morris 2021). This technique enables:

1. High-throughput analysis with tracking of thousands of cells simultaneously, providing a comprehensive picture of cellular dynamics within a tumor.
2. Long-term tracking: DNA barcodes are stably inherited through cell division, allowing researchers to follow cell fate over extended periods.
3. Multiplexing: Different barcodes can be used to label and track distinct cell populations within the same sample, revealing interactions and relationships between them.

In the subsequent sections, we will specifically focus on single-cell viral DNA barcoding studies, discussing their utilization, opportunities, and the technical challenges associated with them.

### Single-cell lineage tracing in developmental biology

With the ability to track the origin and fate of individual cells and their progeny, lineage tracing has been a cornerstone of developmental biology research for decades. scLT with static DNA barcodes has significantly enhanced the resolution in deciphering the complex cellular dynamics and heterogeneity within developing tissues. This advancement surpasses traditional lineage tracing methods, which often provide limited resolution and scalability, typically label a small number of cell populations, and often are restricted to specific cell types (Kebschull and Zador 2018; Bramlett et al. 2020; Wagner and Klein 2020; Chen et al. 2022).

Genetic barcoding was first performed by Jordan and Lemischka (1990), and by Walsh and Cepko (1992), to tag and trace the development of HSCs and the mammalian cerebral cortex, respectively (Serrano et al. 2022). In these early studies, only 100 different retroviral semirandom DNA sequences were used. With the emergence of high-throughput sequencing and the development of new lentiviral-based libraries containing thousands to millions of DNA barcodes, lineage tracing with DNA barcoding has been widely applied in labeling HSCs and tracking their cell fates simultaneously, which greatly enhance the tracing contents and resolution (Gerrits et al. 2010; Lu et al. 2011; Naik et al. 2014). More recently, new libraries that are compatible with scRNA-seq have been developed, which enables systemic evaluation of the relationship between state and fate among millions of cells (Table 1). All these libraries incorporate lineage information within the 3′ UTR of a fluorescence protein transgene to integrate with scRNA-seq.

Weinreb et al. (2020) introduced the LARRY approach to investigate the fate determination map of HSCs using both in vivo mouse models and in vitro cell systems. Employing a “Clone-splitting” strategy, they partitioned barcoded progenitor cells into distinct groups after sufficient expansion and performed scRNA-seq on samples collected along the differentiation trajectory. Based on the continuous transcriptomics landscape, this study uncovered states of primed fate potential of HSCs and two routes of monocyte differentiation leading to mature cells. Additionally, the team developed a computational method to model the dynamic inference of cell fates from single-cell snapshots. However, the study suggested that scRNA-seq fails to capture the heritable properties that guide fate determination, where additional studies such as chromatin accessibility or proteomics information may help to identify the hidden information (VanHorn and Morris 2021). The same research group utilized LARRY in another study to explore clonal trajectories of adult HSCs during long-term bone marrow reconstitution (Rodriguez-Fraticelli et al. 2020). Their findings revealed the existing of an intrinsic molecular signature that characterizes functional long-term repopulating HSCs. Moreover, the study confirmed that the transcription factor TCF15 is required and sufficient to drive HSC quiescent and long-term self-renewal. Beyond exploring the functional aspects of HSCs, this study also established a benchmark for LARRY by assessing long-term clonal tracking in terms of library diversity sufficiency, barcode calling efficiency across various populations, accuracy of single-cell readouts, and minimizing barcode silencing (Rodriguez-Fraticelli et al. 2020). These findings underscored the robustness and reliability of LARRY for studying long-term clonal dynamics in complex biological systems.

The CellTagging approach (Biddy et al. 2018; Kong et al. 2020) was employed to label and track over 100,000 cells. This was achieved through sequential lentiviral delivery of DNA barcodes at different time points to mouse embryonic fibroblasts (MEFs), which enabled layered labeling of these cells. By constructing multilevel lineage trees, the study delineated two paths of fate determination in somatic reprogramming from fibroblasts to endoderm progenitors. Through the comparison of successfully reprogrammed clones and dead-end clones, the investigation identified a candidate gene named *Tmt1a* (also known as *Mettl7a1*). Notably, the addition of this gene to the reprogramming cocktail was found to enhance reprogramming.

By introducing TREX, a system that enables TRacking and gene EXpression profiling of clonally related cells, and Space-TREX, Ratz et al. (2022) studied mouse brain development using in vivo barcode labeling in conjunction with scRNA-seq and spatial transcriptomics. In this groundbreaking study, the team identified fate-restricted progenitor cells in the mouse hippocampal neuroepithelium and showed that microglia originate from a limited number of primitive myeloid precursors that undergo substantial expansion to generate widely dispersed progeny. This study marked the first exploration of migration patterns of clonally related cells at the tissue level, providing insights into understanding tissue architecture in animals through barcode labeling.

### Single-cell lineage tracing in cancer cell origin, metastasis, and drug resistance

The presence of heterogeneous cell populations within tumors, each characterized by distinct genetic and molecular profiles, poses a significant challenge in the development of targeted therapies. This inherent diversity contributes to variations in sensitivity to treatments, complicating efforts to design effective therapeutic strategies. Consequently, it becomes imperative to comprehend and address this heterogeneity for the advancement of cancer treatments. In recent times, DNA barcoding has emerged as a valuable tool in elucidating clonal growth dynamics (Gerrits et al. 2010; Nguyen et al. 2014a, 2015; Porter et al. 2014; Klauke et al. 2015; Belderbos et al. 2017), revealing valuable insights into clone-specific phenotypic behaviors in response to drugs (Bhang et al. 2015; Hata et al. 2016; Lan et al. 2017; Bell et al. 2019; Caiado et al. 2019; Merino et al. 2019; Seth et al. 2019; Feldman et al. 2020), as well as phenomena such as cell plasticity (Lan et al. 2017; Mathis et al. 2017), postsurgery recurrence (Echeverria et al. 2018; Roh et al. 2018; Merino et al. 2019; Rehman et al. 2021), and metastatic potential (Wagenblast et al. 2015; Echeverria et al. 2018; Merino et al. 2019). Besides these studies, the work by Akimov et al. (2020) addressed a critical gap in lineage tracing studies by recognizing the lack of benchmarks to validate clonal dynamics information generated from high-throughput sequencing. To overcome this limitation, the authors employed mixtures of DNA-barcoded cell pools, creating a benchmark read count data set. This data set served as a crucial foundation for statistically inferring differentially responding clones.

Despite the progress in DNA barcoding studies involving targeted DNA-seq for barcode quantification and bulk RNA-seq for gene expression analysis, it is essential to acknowledge that conventional barcode libraries, not compatible with single-cell sequencing platforms, lack the ability to trace clonal diversity at the individual cell level. scLT has emerged as a revolutionary tool in cancer research, allowing the capability to track the fate of individual cells and their progeny over time, and measure the transcriptome of each cell for mechanism study in addition to its clonal identifier (Morgan et al. 2021; Serrano et al. 2022). This approach provides unprecedented insights into tumor heterogeneity, clonal evolution, and the dynamic processes that drive cancer progression.

**Figure 2.**
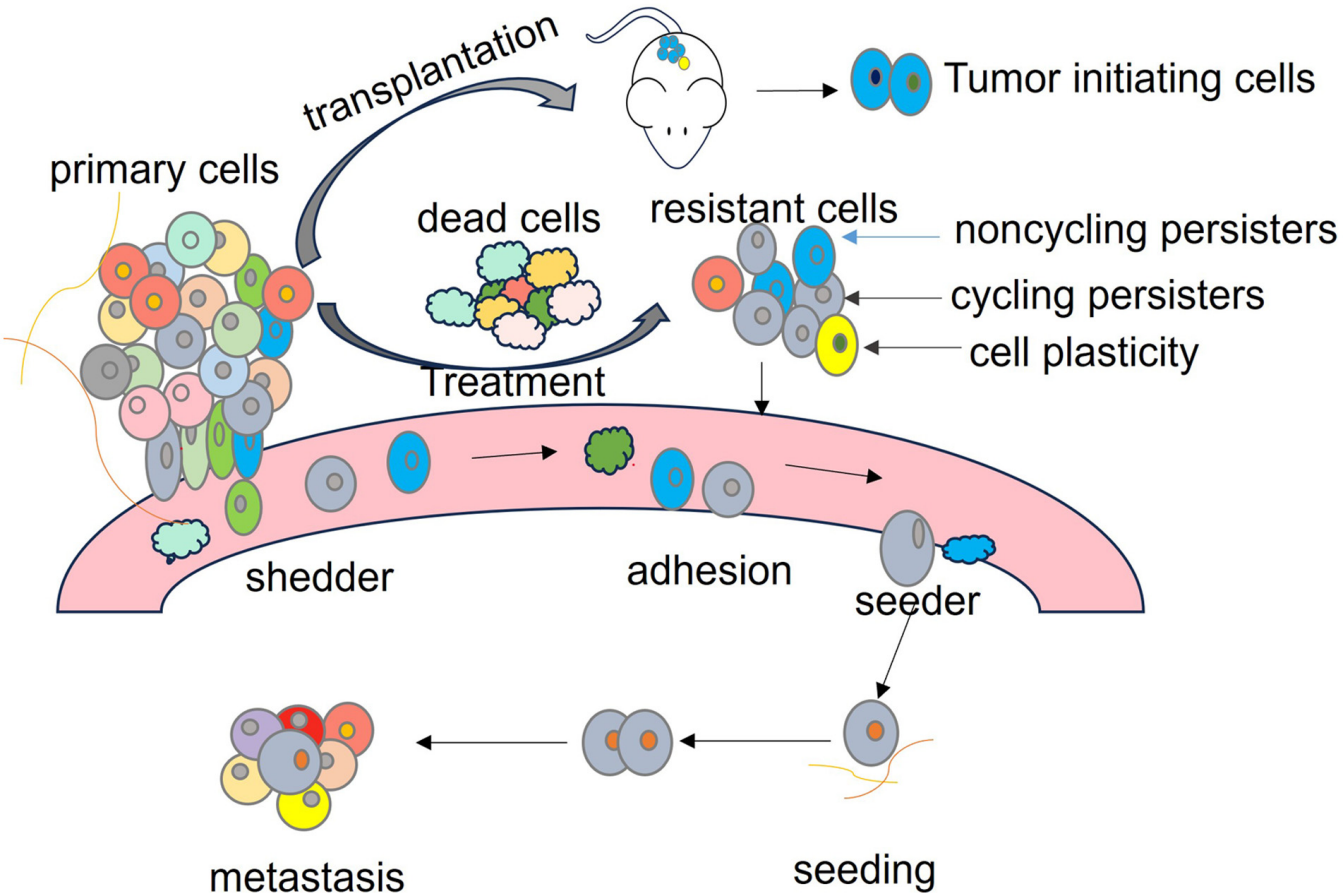
Illustration of tumor heterogeneity summarized based on findings from scLT studies. The primary tumor comprises various cell populations, including tumor-initiating cells capable of initiating a new tumor upon transplantation, metastasis-initiating cells (seeder) with the ability to establish secondary tumors in different sites, drug-resistant cells further classified into cycling persisters (drug-induced transiently resistant proliferative cells) and noncycling persisters (preexisting resistant cells), and drug-sensitive cells susceptible to elimination during treatment. Cell fate is influenced by a combination of genetic and nongenetic information, and it exhibits dynamic changes, known as cell plasticity, under specific circumstances. scLT studies have revealed the existence of “shedder,” “seeder,” “cycling persisters,” and “noncycling persisters.”

Currently, cancer research using scLT is largely focusing on heterogeneous cell behaviors in cancer progression, treatment response, and metastasis, as summarized in Figure 2. It has been applied to investigate markers for melanoma drug resistance (Emert et al. 2021), cancer cell origin and stem cell hierarchies in rhabdomyosarcoma (Wei et al. 2022), clonal dynamics and drug resistance in chronic leukemia lymphoma (CLL) (Gutierrez et al. 2021), clonal behavior in patient-derived xenografts of metastatic triple-negative breast cancer (TNBC) (Merino et al. 2019), cycling persister cells in lung cancer drug resistance (Oren et al. 2021), nonheritable genetic determinants in clonal dynamics within acute myeloid leukemia (AML) (Fennell et al. 2022), and cell plasticity (Table 1; Shlyakhtina et al. 2023).

Emert et al. (2021) introduced Rewind, a novel methodology that integrates genetic barcoding with RNA FISH (fluorescence in situ hybridization) to evaluate rare phenotypic cell events. By employing this approach, they identified *ITGA3* as a novel resistance marker in BRAF600E mutated melanoma cells, through tracing the emergence of vemurafenib-resistant cells back to their naïve counterparts (Emert et al. 2021). Wei et al. (2022) applied the LARRY barcoding system (Weinreb et al. 2020) to label and trace rhabdomyosarcoma cells (multiplicity of infection [MOI] of 0.3). Through the integration with scRNA-seq, they observed that LARRY barcodes were present in 26.4%–47.8% of all scRNA-sequenced cells, with ∼16% of barcodes being shared between parental and daughter cells under various conditions. They concluded that mesenchymal-enriched cells exhibit limited proliferation and possess the capacity to generate cells of diverse states.

Lineage tracing has proven effective in delineating distinct clonal subpopulations in CLL through the utilization of ClonMapper (Gutierrez et al. 2021). This multifunctional barcoding technology seamlessly combines DNA barcoding with scRNA-seq through the expression of gRNA barcodes based on a modified CROP-seq vector and facilitates clonal isolation. This integrated approach enables the identification and characterization of unique clonal subsets based on transcriptomic profiles in CLL. By directly measuring clonal diversification and capturing durable transcriptional signatures of subpopulations, this method retrieved clones from cell cultures before, during, and after treatment (Gutierrez et al. 2021). The study revealed that clones, which were enriched following fludarabine-based chemotherapy, displayed heightened levels of NOTCH, WNT, and CXCR4 signaling in their pretreatment state. In comparison to nonexpanding clones, these enriched clones exhibited a more rapid recovery and enhanced proliferation after the administration of chemotherapy. This highlights the efficacy of the lineage barcode system in tracing developmental dynamics, emphasizing its capability to distinguish and monitor the response of cell clones to therapeutic interventions (Gutierrez et al. 2021; Morgan et al. 2021).

Merino et al. (2019) employed barcoding to unveil complex clonal behavior in patient-derived xenografts of metastatic TNBC. Cells from drug-naive TNBC patient-derived xenograft tumors were barcoded with a lentivirus library containing semirandom DNA barcodes of 98 bp and a GFP reporter. The virus concentration was adjusted to achieve 10%–20% GFP+ in all transfected PDX cells (MOI of 0.1–0.2) (Merino et al. 2019). Barcoded cells were then utilized for clonal assessment both longitudinally, under different conditions, and across multiple tissue sites in mouse models. The study suggested that the majority of disseminated primary tumor cells, “shedder,” lacked the capacity to “seed” in secondary sites, and cisplatin treatment had a minor impact on clonal diversity in the relapsed tumor.

In the Watermelon study (Oren et al. 2021), an mNeonGreen protein was used as a reporter for the DNA barcode, which is inserted on the 3′ UTR of this protein. Similarly, a MOI of 0.3 was utilized. By tracking cells under drug treatment, the study identified a unique proliferative persister lineage (cycling persister) that arises early in the drug treatment process as drug-induced transiently resistant cells. Unlike the majority of persisters (noncycling persisters) in lung cancer cells with *EGFR* mutation undergoing EGFR tyrosine kinase inhibitor (Osimertinib) treatment, this rare lineage not only emerges but also continues to proliferate under drug pressure instead of remaining arrested. scRNA-seq indicated that cycling and noncycling persister cells follow distinct transcriptional trajectories, and cell fate was committed before drug treatment. These findings were further confirmed in other Watermelon models (Oren et al. 2021), including HER2-driven breast cancer and BRAF-driven melanoma and colorectal cell lines.

The SPLINTR (single-cell profiling and lineage tracing) approach was recently employed to investigate nonheritable genetic determinants in clonal dynamics within AML, in which the oncogenic fusions of the *MLL1* gene is identified as a key driver of aggressive malignancy (Fennell et al. 2022). Through the application of sequential labeling with expressed DNA barcodes, coupled with scRNA-seq, the study revealed that the dominance of malignant clones is intrinsically tied to the cell. This heritable property is facilitated by the repression of antigen presentation and the increased expression of the secretory leukocyte peptidase inhibitor (*Slpi*) gene, which was genetically verified in the study. The research further demonstrated that increased transcriptional heterogeneity plays a crucial role in enabling clonal fitness across diverse tissues and immune microenvironments, as well as in the context of clonal competition. These insights into nongenetic transcriptional processes provide valuable information that may shape future therapeutic strategies for AML (Fennell et al. 2022).

BdLT-Seq, as employed in a study by Shlyakhtina et al. (2023), represents a novel approach for studying cell plasticity over extended periods of cell culture. In contrast to commonly used lentivirus DNA barcode libraries, this study introduced a library of engineered episomes. Each episome carries a unique barcode, which is encoded in the 3′ UTR of a reporter gene (Shlyakhtina et al. 2023). These episomes have the advantage of being stably maintained and expressed within transfected cells, facilitating both short- and long-term lineage tracing. However, a distinctive feature of this system is the random inheritance of episomes by daughter cells during cell division. This random inheritance results in a decay in the number of uniquely barcoded episomes present in cells downstream from any given lineage (Shlyakhtina et al. 2023). This distinctive barcode-based fingerprint offers a valuable tool for exploring and understanding cell lineage dynamics and plasticity over extended periods of cell culture. This study revealed insightful findings, indicating that cell transcriptome states are not only inherited but also dynamically reshaped based on constrained rules encoded within the cell lineage. These were observed under various conditions, including basal growth conditions, upon oncogene activation, and throughout the process of reversible resistance to therapeutic cues. Importantly, this dynamic reshaping of cell transcriptomes allows for the adjustment of phenotypic output, leading to intraclonal nongenetic diversity (Shlyakhtina et al. 2023).

The development of drug resistance plays a pivotal role in cancer therapy. To address this challenge, Johnson et al. (2020) constructed a mathematics framework aimed at predicting the responsiveness of cells to treatment utilizing snapshots of lineage-traced scRNA-seq data. Employing the COLBERT (Control of Lineages by Barcode Enabled Recombinant Transcription) barcoding system (Al'Khafaji et al. 2018) to uniquely tag and longitudinally track the cells, the researchers identified clones that exhibited significant decreases or enrichments after treatment. Subsequently, a classifier was developed based on the pretreatment transcriptomics of these identified cells. This classifier was then utilized to estimate the phenotypic composition of cells at various time points during the treatment response. This mechanistic model proposed incorporated inputs such as the relative fractions of different phenotypes and distinct longitudinal measurements of cell numbers, providing a comprehensive basis for predicting therapeutic responses or resistance. This innovative methodology contributes to advancing our understanding of cancer treatment dynamics and holds promise for enhancing the efficacy of therapeutic interventions.

In summary, the highlighted scLT studies reveal substantial heterogeneity among cancer cells, encompassing distinct subtypes such as tumor-initiating cells, metastasis-initiation cells, drug-sensitive cells, and drug-resistant cells as illustrated in Figure 2. These insights, which are often not achievable with traditional methods, underscore the complexity and diversity of cancer cell behaviors within tumors. Within the drug-resistant category, cells can be classified as cycling persister cells and noncycling persister cells. Moreover, cell plasticity allows for dynamic transitions in cell stage and phenotype in response to different conditions. The determination of cell fate and behaviors involves a complex interplay of both genetic and nongenetic information. The intricate interplay of various cell types and their dynamic responses to therapeutic interventions poses ongoing challenges in fully understanding and effectively treating cancer. Continued research efforts and advancements in technologies like scLT are essential for refining our comprehension of cancer heterogeneity and devising targeted therapeutic strategies to combat this complex disease.

## Single-cell lineage tracing experiments

**Figure 3.**
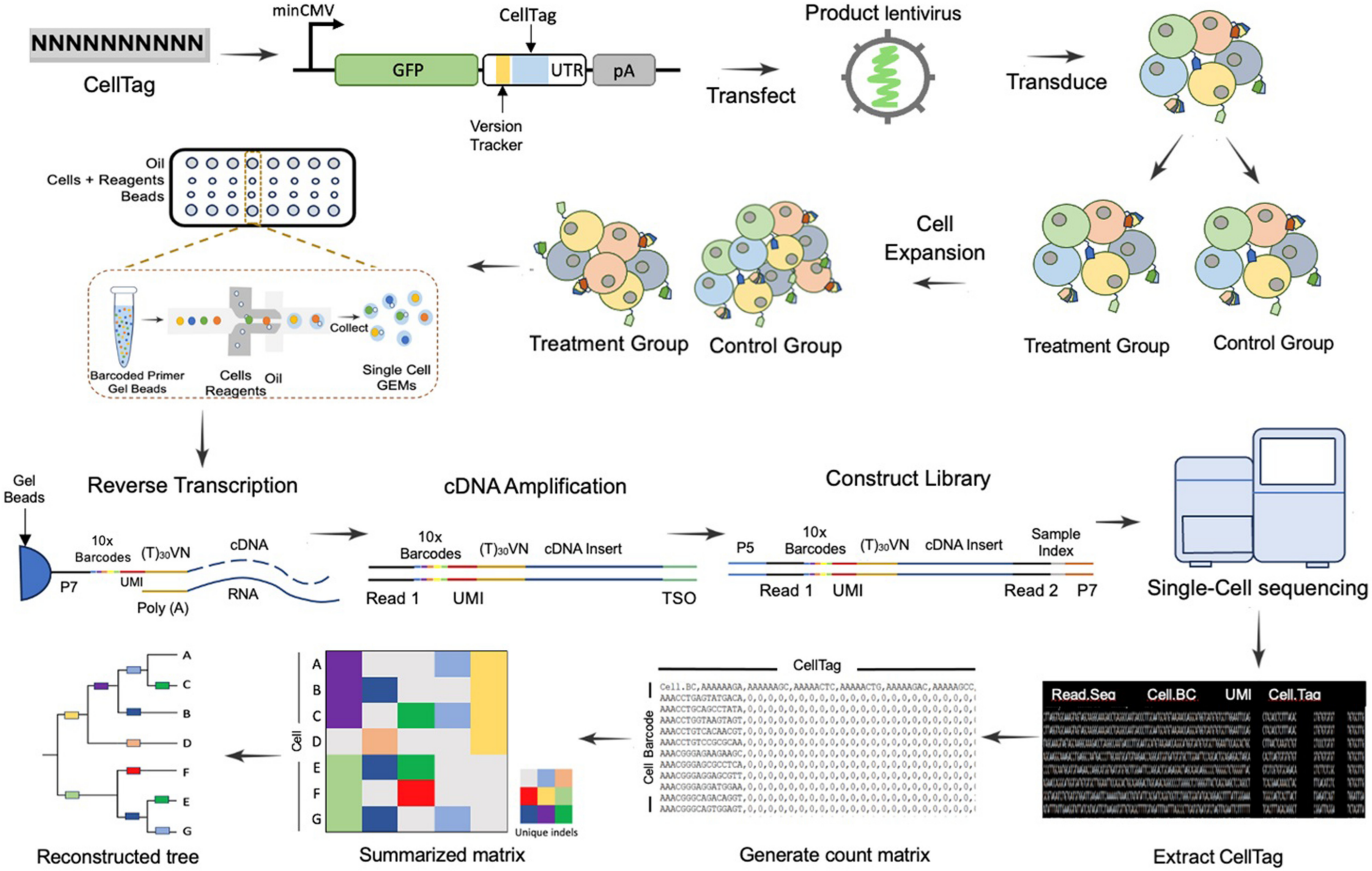
Standard workflow of single-cell lentivirus barcoding lineage tracing experiment. *Lentivirus barcode labeling*: cells of interest are labeled with DNA barcodes through lentivirus transfection. *Cell expansion and splitting*: After reaching the required cell numbers, cells are split for drug treatment or challenges, with one portion saved as a parental control. *Single-cell RNA sequencing (10x Genomics)*: 10x Genomics is employed for scRNA-seq, where CellTag sequence can be captured from the 3′ UTR of fluorescence gene similar to other genes. This step involves Gel Bead Emulsions, reverse transcription, amplification, and sequencing. *Computational analysis*: DNA barcodes are used in computational analysis to connect cell fate after treatment or challenge to its origin based on clone calling and lineage tree inference. For example, the summarization matrix and the lineage tree show that cells A, B, and C have the same barcodes and they are inferred to have a closer relationship from lineage analysis, so do cells E, F, and G. *Molecular mechanism investigation*: Single-cell transcriptomics data are utilized to understand the molecular mechanisms underlying fate determination.

Experimental design plays a crucial role in scLT experiments as it directly impacts the accuracy, reliability, and interpretability of the obtained results. Factors such as the selection of the barcode system, the method of barcode introduction, and the inclusion of appropriate controls have a significant impact on the ability to accurately capture and analyze clonal dynamics. Crucial considerations in barcoding studies encompass barcode library diversity, the experimental timeline, starting cell numbers, MOI, barcode stability, cell culture conditions, scRNA-seq techniques, quality control, filtering, clone calling, and barcode recovery, etc. Thoughtful attention to these factors ensures the success and accurate interpretation of lineage tracing experiments. Rigorous experimental design is particularly essential when unraveling complex biological phenomena, including the identification of rare subpopulations, the assessment of clonal diversity, and the exploration of dynamic cellular behaviors. In the following sections, we will discuss the key factors contributing to a successful single-cell viral DNA barcoding experiment. A typical design and workflow of a single-cell viral DNA barcoding experiment is depicted in Figure 3.

### DNA barcode library diversity

The most employed DNA barcodes typically consist of a fluorescent protein tag such as GFP followed by a DNA segment with varying numbers of nucleotides. The florescence signal is used to indicate the presence of barcodes and to evaluate the transduction efficiency. These barcodes are integrated into lentivirus plasmids for efficient delivery and expression within target cells (Figs. 1, 3). When designing the DNA sequence, it is crucial to consider a balance—long enough to ensure a diverse library based on the theoretical size of 4^*N*^ (*N* = number of nucleotides), but not too long to surpass sequencing capacity (Bramlett et al. 2020). A longer DNA segment correlates with a larger diversity of the barcode library, which enhances the ability to uniquely label a greater number of cells. However, this increased length can result in higher costs for library synthesis and pose sequencing challenges, such as elevated sequencing errors and data complexity. Unlike the majority of DNA barcode tracing studies with random DNA sequences, which typically employ barcode lengths ranging from ∼20 to 32 bp (Table 1), the CellTagging library utilizes DNA barcodes that are notably shorter, with a length of only 8 bp (Biddy et al. 2018). Semirandom barcode libraries like SPLINTR (Fennell et al. 2022) typically use longer DNA sequences as shown in Table 1.

Before introducing the lentiviral barcode library into cells of interest, it is imperative to evaluate viral titer and barcode diversity in cell lines. This involves transduction, barcode extraction, and sequencing. The sequencing results establish reference libraries for downstream bioinformatics analysis (Bramlett et al. 2020). Library diversity is essential to minimize the likelihood of labeling more than one cell with the same barcode. Ideally, each barcode should represent a single-cell clone, and the library's diversity determines the number of unique clones trackable in a single experiment. Therefore, optimizing the transfection step, including the starting cell number and viral titer, is crucial for enhancing barcode diversity (Bramlett et al. 2020). Bramlett et al. (2020) suggested that a library of 40,000–50,000 barcodes typically allows tracking of ∼1000 cells with a >95% probability, i.e., >95% of the barcodes represent single cells. Barcode libraries that are compatible with scRNA-seq, such as LARRY (Weinreb et al. 2020), ClonMapper (Gutierrez et al. 2021), and Watermelon (Oren et al. 2021) normally contain millions of unique barcodes, allowing for labeling and tracing thousands even millions of cells simultaneously (Morgan et al. 2021; VanHorn and Morris 2021; Serrano et al. 2022).

### Viral barcoding scLT cell culture parameters

In a standard lineage tracing experiment, cells are infected with lentivirus barcodes (Table 1; Fig. 3). Following infection, typically after 48–72 h to allow for barcode integration, cells are subjected to FACS or antibiotic selection processes to isolate barcoded cells. The choice between these selection methods depends on the specific requirements of the experiment. While FACS enables the isolation of cells based on their fluorescence reporter transgene expression, antibiotic selection provides a convenient and efficient means to select cells with integrated barcodes. It is worth noting that antibiotic selection may introduce additional stress to cell growth, and as a result, the use of a fluorescence reporter transgene is a common preference, minimizing potential adverse effects on cellular health during the isolation process.

Most published studies chose a very low MOI, typically ranging from 0.1 to 0.5. This low MOI ensures that each cell receives only one unique barcode. This strategic approach enhances the clarity and unambiguity in identifying and tracing clone relationships within the experimental setup. In contrast, studies using the CellTagging approach used a high MOI of 3–4 to allow for multiple barcode combinations (Biddy et al. 2018; VanHorn and Morris 2021). The initially sorted barcoded cells determine the initial barcode diversity and sample clonality. This step is crucial for isolating cells that have successfully incorporated the barcodes, facilitating the subsequent analysis, and investigation of their unique characteristics or responses. These cells are then allowed to proliferate to attain sufficient representation of each individual cell. Subsequently, the cell pool is divided into multiple samples using the “Clone-splitting experiment” approach (Serrano et al. 2022), with one portion designated as the parental control to establish baseline barcode representation. The remaining samples are used for specific treatments or challenges, such as drug administration or xenografting. At the conclusion of the experimental period, the treated cells are harvested and prepared for scRNA-seq along with the baseline control cells (Fig. 3). The barcodes present in these samples play a pivotal role in linking the clones back to their cellular origin. This linkage allows for the establishment of clonal relationships, thereby enabling the investigation of clonal differences, heterogeneity, or responses to diverse challenges at a single-cell resolution.

Presently, the widely adopted scRNA-seq technology is the 10x Chromium 3′ kit (Table 1). However, the sequencing capacity of this technology is limited to ∼10,000 cells per sample. Additionally, the high cost associated with scRNA-seq poses a limitation on the number of samples that can be feasibly sequenced to cover the barcode diversity within a single experiment. This limitation underscores the importance of carefully determining the initial cell count, MOI, cell cycle, culture duration, and splitting intervals in experimental design. The definition of MOI and explanation of how the number of cells with a positive DNA barcode is calculated based on the MOI and the initial number of cells is illustrated in Box 1. With the combination of these factors, we want to ensure the number of tracked cells is sufficient to capture the diversity of the population and the dynamics of lineage progression. If the number is too low, we may miss important cellular events or rare lineages.

Box 1. **Definition of MOI and calculation of barcode positive cell numbers**

MOI is defined as the ratio of infectious viruses to cells in a cell culture. In the case of DNA barcoding, it indicates the number of unique barcodes in a specific infected cell. Assuming the number of lentiviruses (barcodes) infecting a cell follows a random distribution, the number of viruses infecting each cell can be calculated from the Poisson distribution:

$$P(n)=\frac{(m^{n}\;\times \;e^{-m})}{n!}$$

where *P*(*n*) is the probability that a cell will be infected with exactly *n* viruses, and *m* is the average number of viruses per cell (i.e., MOI) (Shabram and Aguilar-Cordova 2000). If we infect 1 million cells at an MOI of 0.1 in DNA barcoding, we would expect that *P*(0) = e^−0.1^ = 90.5% of cells are not infected, *P*(1) = 0.1 × 0.9 = 9.05% of cells have 1 viral particle, *P*(>1) = 0.45% of cells have >1 viral particles. Therefore, an MOI of 0.1 enables over 95% of labeled cells having a unique barcode.

Inversely, the number of cells with a positive DNA barcode can be estimated using the following formula:

$$K_{\mathrm{positive}}=N_{\mathrm{initial}}\times (1-e^{-m})$$

where *K*_positive_ is the number of positively barcoded cells, *N*_initial_ is the initial number of target cells, and *m* is the MOI.

For instance, if we start with 50,000 cells and use an MOI of 0.1, the expected number of cells with unique positive DNA barcodes can be calculated as follows:

$$K_{\mathrm{positive}}=50,000\times (1-e^{-0.1})\approx 50,000\times 0.0952\approx 4760$$

Considering the heterogeneity of cell fates within the population, tracking a larger number of cells may be necessary to comprehensively understand lineage dynamics. In scenarios involving rare events, such as the study of tumor-initiating cells or preexisting drug-resistant cells, it becomes crucial to ensure an ample starting cell population. This precaution is necessary to contain a sufficient number of these rare cells, considering that studies across various cancer types have reported the percentage of these rare cells ranging from <1% to ∼20% (Serrano et al. 2022). As illustrated in Box 1, if we employ a MOI of 0.1 to infect 50,000 cancer cells, we can anticipate obtaining ∼4700 cells with unique positive DNA barcodes after the initial cell sorting. Within this population, there would be an expected presence of ∼47–940 rare preexisting drug-resistant cells. To set up three treatment conditions for studying cell responses, a potential strategy involves allowing the initially labeled cells to proliferate for approximately four generations, reaching ∼16 cells per clone and a total of ∼75,000 cells. Subsequently, the cells can be split into four samples (∼19,000 cells per sample and approximately four replicates per clone), with one sample serving as a baseline control to assess cell states and barcode diversity using 10x scRNA-seq. 19,000 cells may require two 10x sequencing runs to cover all the cells adequately. However, considering potential cell loss during subsequent preparation steps such as further cell sorting and later droplet encapsulation for sequencing, it is reasonable to conclude that one single sequencing lane may be sufficient to capture the clonal information from the entire cell population. This careful consideration ensures cost-effectiveness while maintaining the robustness of the sequencing results.

Following treatment, an anticipated outcome is a significant reduction in the number of unique barcodes. This reduction provides a means to track the clones that have undergone various treatments back to the baseline control, enabling the identification of rare cells that may have sustained different treatment conditions. This approach not only allows for the assessment of barcode diversity but also facilitates a comprehensive investigation of cell responses and associated molecular mechanisms under distinct treatment conditions. By comparing the barcode profiles posttreatment to the baseline, researchers can gain valuable insights into the behavior and dynamics of rare cell populations in response to specific challenges as demonstrated in previous studies (Emert et al. 2021; Fennell et al. 2022; Shlyakhtina et al. 2023).

### Clone calling in scLT

In current scLT technologies, the capture of DNA barcode sequences typically relies on 3′ end single-cell sequencing. In studies utilizing LARRY, scRNA-seq is employed with a customized procedure called inDrops that includes a specific step to amplify barcodes containing mRNA transcripts (Rodriguez-Fraticelli et al. 2020; Weinreb et al. 2020). However, other studies (Biddy et al. 2018; Fennell et al. 2022; Ratz et al. 2022; Shlyakhtina et al. 2023) employing the 10x Chromium 3′ kit are not compatible with this specific amplification step in their procedures, as the kit's design may not allow for such customization. Unlike in the customized sequencing where the enriched barcodes have a higher probability of being captured, in standard 10x Chromium sequencing, the probability of capturing the DNA barcode inserted in the 3′ UTR of the fluorescence gene is similar to that of other genes. This likelihood depends on the expression abundance of the fluorescence gene within a specific cell. In both the customized sequencing and 10x 3′ Chromium single-cell 3′ scRNA-seq, individual cells are emulsified with Gel Beads to form GEMs (Gel Beads in Emulsions) during library preparation (Fig. 3). Each GEM contains a single cell, a single Gel Bead, and the reverse transcriptase reagents. GEMs are generated in parallel within the microfluidic channels of the chip, allowing for the simultaneous processing of hundreds to tens of thousands of single cells. Within each GEM reaction vesicle, a single cell undergoes lysis, the Gel Bead is dissolved to release identically barcoded reverse transcriptase oligonucleotides into solution, and reverse transcription of polyadenylated mRNA occurs. Consequently, all cDNAs from a single cell will share the same barcode (Cell-BC), facilitating the mapping of sequencing reads back to their original single cells of origin. In addition to the cell barcode, molecules in each cell also are tagged with a unique molecular identifier (UMI). The UMI serves as a unique tag for individual mRNA molecules, allowing for precise quantification of gene expression within a specific cell. In addition, the barcoded cells will have a unique lineage barcode (Lineage-BC), allowing lineage tracing between offspring and parental cells.

**Figure 4.**
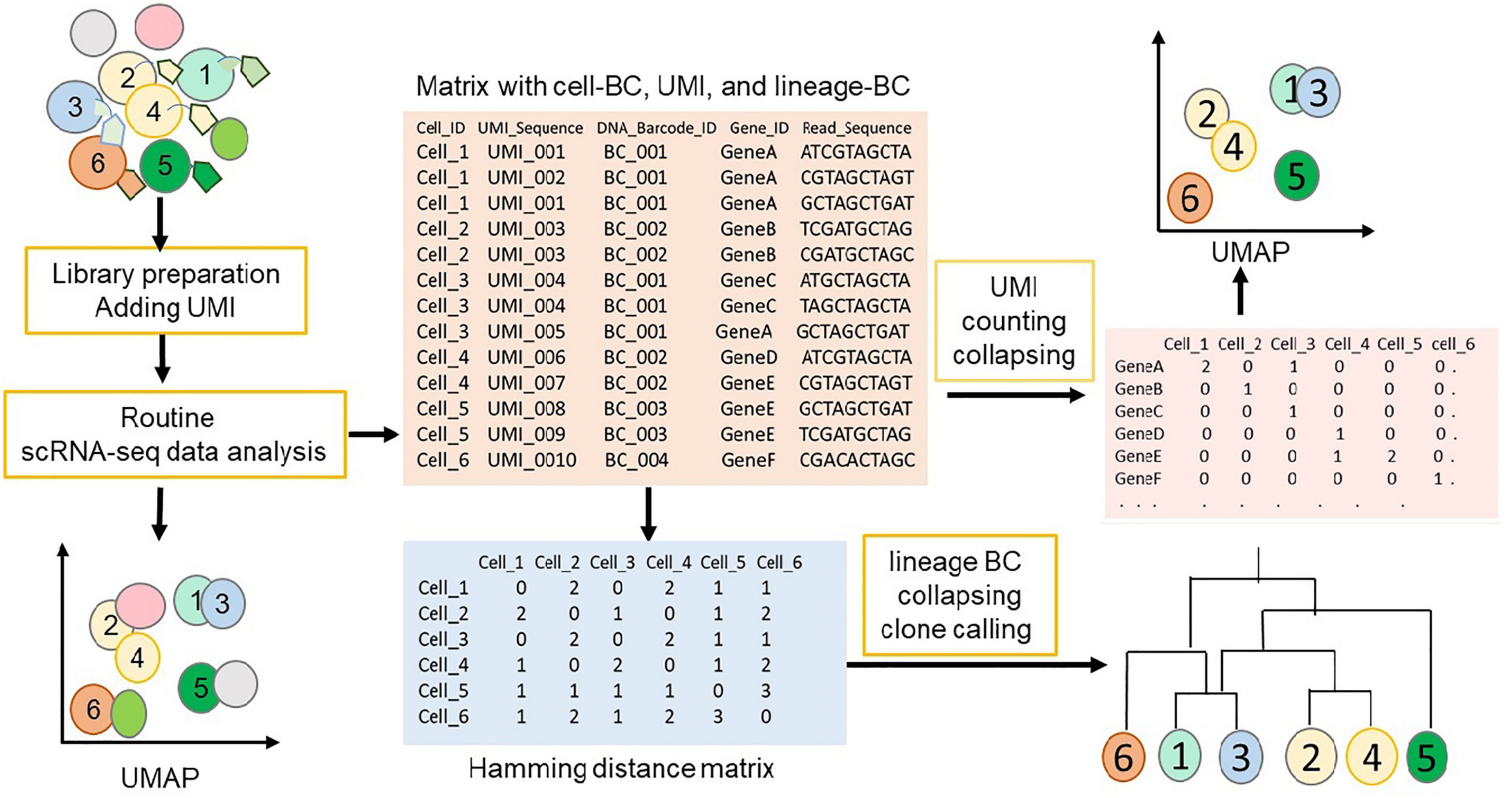
Overall steps in scLT data analysis and barcode calling. Before scRNA-seq library preparation, only cells 1–6 were labeled with lineage barcodes. scLT data can first be analyzed using routine scRNA-seq data analysis steps, such as read alignment, UMI counting and collapsing, filtering, normalization, and clustering, including cells with and without lineage barcodes. Then, the data can be filtered based on UMI counts and lineage barcode availability to obtain cells with cell barcodes, UMIs, and lineage barcodes. This filtered data can be further analyzed using regular scRNA-seq steps or used for lineage analysis to construct lineage trees based on cell similarity determined by Hamming distance or other methods.

In published scLT studies, researchers often describe customized pipelines for the analysis of scRNA-seq data, especially for clone calling. These customized pipelines are tailored to the specific experimental design, the characteristics of the data generated, and the objectives of the study. Figure 4 and Box 2 demonstrate and explain the general steps in scLT data analysis and clone calling.

Box 2. **ScLT data analysis and cloning calling**

The first part of the analysis in scLT is similar to traditional scRNA-seq analysis before lineage barcode calling and clone calling. They typically encompass steps such as quality control, sequence alignment, barcode demultiplexing, UMI counting, normalization, filtering, dimensionality reduction, and clustering of gene expression data to identify different cell types and states. After this initial analysis, the lineage barcodes can be identified and analyzed to reconstruct cell lineage and clonal relationships. In the process of calling lineage barcodes in scLT studies, cells containing information on all triples (Cell-BC, UMI, and Lineage-BC) are extracted. To ensure the unambiguity of assigning Lineage-BC to cells, it becomes critical to establish a cutoff for UMI read counts at this stage. This cutoff helps filter out cells with insufficient UMI counts, thereby enhancing the reliability of lineage barcode assignments. After lineage labels are assigned to cells, the next step involves collapsing or aggregating cells with the same Lineage-BC. Hamming distance or Jaccard distance with a threshold that measures the similarity of two cells has been commonly used for collapsing (Rodriguez-Fraticelli et al. 2020; Weinreb et al. 2020; Weng et al. 2024). Cells within the same lineage whose Hamming or Jaccard distance falls below the chosen threshold can be grouped together. These groups represent cells that are considered similar in terms of their gene expression profiles and are collapsed into a single representative cell.

In clone calling for scLT, assigning cells with the exact same set of barcodes as clones is crucial. This underscores the importance of ensuring, during the initial infection step, that each cell receives only one unique barcode. As described by Weinreb et al., it is essential to discard cell pairs with the same Cell-BC and Lineage-BC from different sequencing libraries. Additionally, attention should be paid to clones that exhibit overdominance within a single sequencing library compared to other sequencing libraries of the same sample (Rodriguez-Fraticelli et al. 2020; Weinreb et al. 2020). To quantify clone similarity in clone calling, various metrics such as Hamming distance (Rodriguez-Fraticelli et al. 2020; Weinreb et al. 2020; Ratz et al. 2022), Jaccard index (Biddy et al. 2018; Weng et al. 2024), Pearson's correlation, or clustering algorithms (Fennell et al. 2022) have been reported. These metrics help assess the similarity or dissimilarity between clones, providing a quantitative basis for identifying and characterizing clonal relationships in scLT studies. During barcode calling, the background collision rate is an important factor to consider (McKenna et al. 2016; Weinreb and Klein 2020; Weng et al. 2024). It refers to the probability that two or more distinct cells will be mistakenly assigned the same DNA barcode purely by chance, rather than due to a true biological lineage relationship. This can occur when the diversity of the barcodes is insufficient to uniquely label each cell, especially when the number of cells exceeds the number of unique barcodes available. Therefore, barcode diversity, the number of starting cells, and sequencing depth are crucial factors to mitigate the risk of collision and ensure accurate lineage tracing.

The results obtained from computational methods and analyses in lineage studies should be rigorously validated. Validation is a critical step in confirming the accuracy of lineage tracking results. This involves benchmarking against known data sets or experimental controls. By comparing the outcomes of the computational methods with established ground truth information or controlled experimental conditions, researchers can assess the reliability of the identified clonal relationships and validate the effectiveness of their lineage-tracking approach. Validation not only ensures the accuracy of the results but also enhances the confidence in the interpretation of clonal dynamics and relationships within the studied biological system. It is an essential component of the scientific rigor required in scLT studies. However, the novelty of scLT technology indeed poses challenges when it comes to establishing standard training data sets or benchmarking approaches. In many cases, data sets with a clear “ground truth” for clonal relationships may not exist, making validation a complex task (VanHorn and Morris 2021). Recently, new pipelines are emerging to reconstruct lineages from a single round of barcoding (Johnson et al. 2020; Weinreb and Klein 2020). More novel methods are required for robust and standard analysis in future scLT studies.

### Barcode recovery in viral barcoding scLT

Barcode dropout represents a critical challenge in lineage tracing, and various factors during the experimental process can contribute to this issue (Bramlett et al. 2020). First, the barcode dropout can be attributed by temporal dynamics of barcode stability and FACS sorting. Our experience indicates that the integration of DNA barcodes tends to stabilize after 2–3 weeks after transduction. By the third week, we noticed that ∼90% of the cells initially identified as positive during sorting at 48–72 h retained their positivity. This emphasizes the importance of further sorting before splitting samples, while FACS sorting could lead to a further reduction in labeled cell numbers. Therefore, it becomes crucial to account for these factors when estimating cell numbers for scRNA-seq to ensure accurate and reliable results in downstream analyses. Second, barcode drop out could be caused by silencing or low expression of the barcodes, which limits the detection by scRNA-seq that relies on the capture of expressed barcodes. This partial detection of the barcodes is a particular issue when multiple, independent barcodes are needed to comprise a complete lineage label (VanHorn and Morris 2021). Third, cell division may cause unequal distribution of barcodes among daughter cells that result in the loss of barcodes in some cells, contributing to barcode dropouts and reduced recovery rates.

Another crucial factor contributing to barcode dropout is the labeling and capture rate of cells within a population, especially in the context of in vivo studies. Cell death or inadequate cell dissociation can lead to failures in cell capture (VanHorn and Morris 2021). As illustrated in the TREX study (Ratz et al. 2022), only 0.51% of all initially barcoded cells were found to be present in the tissue when Space-TREX was applied for spatial high-density clonal tracking in mouse brain tissue. This significant loss was attributed to multiple stages in the experimental process, including the loss during tissue dissociation (10.6% of cells recovered), FACS sorting (35%–64% of sorted cells recovered), droplet encapsulation (50% of loaded cells recovered), as well as cloneID dropout from a subset of sequenced cells (24%–51% containing a cloneID) (Ratz et al. 2022). The study also provided a summary of barcode recovery rates from various scLT studies, revealing a broad spectrum of recovery rates ranging from 11% to 74%. Additionally, Biddy et al. (2018) reported that the expression of CellTag is lost in 11 ± 2% of cells by day 28. This wide variability underscores the diversity of experimental conditions, methodologies, and challenges associated with barcode recovery in different research contexts. This diversity highlights the complexity of factors influencing the success of scLT experiments. Consequently, it emphasizes the critical need for careful consideration and optimization in experimental design to ensure reliable and meaningful results across studies (Ratz et al. 2022).

The barcode recovery rate is significantly linked to the probability of identifying a clone at different time points or under different treatment conditions. Weinreb et al. (2020) outlined three key factors influencing this probability for *N* initially barcoded cells:

1. *P*(split): the probability that members of the same clone are physically present in both fractions when cells are split for sequencing and replating.
2. *P*(detect early): the probability that cells in the fraction designated for immediate sequencing are actually detected.
3. *P*(detect late): the probability that cells in the replated fraction survive cell culture/treatment and appear in the late time point data set.

The final yield of labeled cells is then proportional to *N* × *P*(split) × *P*(detect early) × *P*(detect late). Balancing the initial cell numbers for barcoding becomes a critical consideration to avoid an excessive number of clones that exceed the single-cell sequencing capacity, ensuring that *P*(detect early) × *P*(detect late) does not reach low values (Weinreb et al. 2020).

However, these parameters may not be intuitive during the planning phase of scLT studies. Hence, optimizing initial cell numbers, culture duration, sample splitting time, and related sample collection time through pilot studies is critical and highly encouraged to enhance barcode recovery, as well as the reliability and informativeness of scLT experiments. This iterative optimization process ensures that the experimental design aligns with the goals of the study and maximizes the chances of successful barcode detection.

## Summary and future perspectives

Prospective genetic lineage barcoding technologies have found extensive application for simultaneously tracking and analyzing clonal relationships in populations ranging from hundreds to millions of cells. The choice of barcode type depends on the specific objectives of the study. Typically, viral integration barcoding offers a vast barcoding space, allowing for the simultaneous labeling of thousands to millions of clones through early barcoding. Through the integration of clonal relationships and single-cell transcriptomics, scLT with viral barcoding have demonstrated remarkable precision in uncovering cell lineages and clonal dynamics in the realms of developmental biology and cancer heterogeneity. Despite these advantages, there are still challenges associated with this cutting-edge technology.

Firstly, the occurrence of barcode dropouts and a low recovery rate poses limitations on the precision of lineage reconstruction. This can be attributed by technical limitations in barcode insertion and detection methods, genetic heterogeneity among cells leading to variations in barcode expression efficiency, unequal segregation of barcodes during cell division, and biological noises, among other factors. To address these challenges, novel research focusing on refining experimental techniques, improving the design of barcoding systems, and developing computational methods to mitigate the impact of dropouts and enhance the reliability of scLT is highly needed. Advances in technology and a deeper understanding of these factors are crucial for overcoming these limitations and improving the accuracy of lineage reconstruction in single-cell studies. For example, to address challenges such as insufficient expression of barcodes in specific cells, an effective strategy may involve integrating a single-cell multiomics approach, such as G&T-seq. This innovative technique allows for the simultaneous detection of DNA and RNA at a genome-wide scale within the same cell. Embracing such an approach holds great promise in enhancing barcode detection rates and overcoming limitations associated with inadequate transgene expression in certain cells.

Besides the high dropout rates, another challenge in prospective scLT is the ability to trace cells throughout a long period, either with one-time or multiple instances of static barcode insertion or with the continuous generation of new barcodes. Most studies using static barcodes add these barcodes at the initial starting time point. This approach involves introducing a unique DNA barcode or barcode combination into each cell at the beginning of the experiment, which remains unchanged as the cells proliferate and differentiate over time (Rodriguez-Fraticelli et al. 2020; Weinreb et al. 2020; Gutierrez et al. 2021; Fennell et al. 2022; Ratz et al. 2022). However, this method has limitations in long-term studies, as it does not capture dynamic changes in cell lineage or allow for the identification of new cell populations that emerge later. Several studies have introduced additional static DNA barcodes to label the initially labeled cells at a later time point, such as in studies performed using CellTagging (Biddy et al. 2018; Kong et al. 2020). However, selecting the right time or interval for later time tagging is a significant challenge (Chen et al. 2022). The timing of barcode introduction is crucial because it needs to be aligned with specific biological events of interest, such as the onset of differentiation, response to a treatment, or emergence of a particular cell population (Wagner and Klein 2020). Tagging too early may miss critical later events, while tagging too late might not capture the early lineage relationships. Therefore, optimizing the timing for adding DNA barcodes at a later time point for an additional layer of cell tracking requires a deep understanding of the biological system and careful experimental design to ensure that the most informative stages are captured. In contrast, methods that continuously generate new barcodes throughout the experiment can provide a more detailed and dynamic picture of cell lineage relationships. These approaches, such as CRISPR-based lineage tracing (Alemany et al. 2018; Spanjaard et al. 2018; Chan et al. 2019; McKenna and Gagnon 2019; Quinn et al. 2021; Lin et al. 2023), introduce new mutations at regular intervals, creating a more complex and informative barcode pattern that reflects ongoing cellular events. This continuous barcoding can help track cell divisions, migrations, and differentiation processes more accurately, but it also introduces additional complexity in data analysis and interpretation.

A third challenge in the field is the absence of standardized benchmarking data sets and computational methods, complicating the validation of scLT results. The novelty of this technology presents difficulties in establishing clear benchmarks or ground truth data for assessing accuracy. Many studies involving viral integration barcodes develop custom pipelines tailored to their specific data. For example, Kong et al. (2020) built an analytical pipeline to study lineage hierarchies for their barcoding technique called CellTagging, which employed several rounds of lentivirus infections to achieve sequential barcoding. Weinreb and Klein (2020) developed a pipeline for analyzing lineage barcoding experiments in hematopoiesis. LineageOT was developed for inferring developmental trajectories from snapshots of both cell lineage and cell state (Forrow and Schiebinger 2021), and Cospar was developed to study clonal dynamics (Wang et al. 2022). Unfortunately, these customized in-house pipelines are often user-unfriendly, hindering the ability to compare results across different studies. This limitation constrains the broader expansion of barcoding and clonal tracking experiments (Lyne et al. 2018). In response to this challenge, two R-based programs, i.e., genBaRcode (Thielecke et al. 2020) and barcodetrackR (Espinoza et al. 2021), have been introduced to establish standardized data analysis procedures. GenBaRcode was developed to facilitate routine barcode data analysis by offering features such as barcode sequence identification, abundance quantification with error correction, and visualization functions. Moreover, it provides a user-friendly graphical user interface, catering to those less experienced in R, to conduct analyses effectively (Thielecke et al. 2020). BarcodetrackR incorporates a range of tools designed for the comprehensive analysis and visualization of clonal tracking data, especially for exploring longitudinal clonal patterns and lineage relationships in clonal tracking studies involving hematopoietic stem and progenitor cells (HSPCs) (Espinoza et al. 2021). It is important to note, however, that neither program was developed for scLT data. Fueled by the CRISPR–Cas9 genome editing technology, tremendous recent advances in computational program development have been observed for CRISPR-based scLT, focusing on tree reconstruction. Some programs are based on observed edited barcodes only, such as distance-based DCLEAR (Gong et al. 2022), machine-learning-based AMbeRland (Gong et al. 2021; Chen et al. 2022), maximum-parsimony-based Cassiopeia (Jones et al. 2020), and Startle (Sashittal et al. 2023). Some recent programs can simultaneously integrate lineage tracing and transcriptome data for lineage tree inference, such as neighbor-joining and maximum-likelihood-based LinRace (Pan et al. 2023). Additionally, the maximum-likelihood-based framework LinTIMaT (Zafar et al. 2020) can integrate both mutational and transcriptional data for reconstructing lineage trees. Despite these advances, there is a considerable demand for computational programs tailored to ensure standardization and robust analysis of scLT data, especially for integrating DNA barcode data analysis.

Finally, the existing scLT methods are primarily designed for compatibility with scRNA-seq platforms, and they often lack the capacity to capture spatial information. This limitation hinders a comprehensive understanding of complex gene regulatory networks at the single-cell level and impedes the exploration of the spatial context in which clonal dynamics unfold within tissues or organs. The development of novel barcode libraries holds the promise of enabling barcode detection in single-cell multiomics and spatial transcriptomic data sets. In a recent study, Jindal et al. (2023) applied CellTag-multi, a technique for capturing heritable random barcodes expressed as polyadenylated transcripts in both scRNA-seq and single-cell Assay for Transposase Accessible Chromatin sequencing (scATAC-seq). This method enables independent clonal tracking of transcriptional and epigenomic cell states. Additionally, Ratz et al. (2022) developed Space-TREX, a method grounded in spatial transcriptomics, facilitating the concurrent profiling of spatial gene and protein expression along with clonal barcodes in the same tissue section. These studies mark a significant advancement, opening new avenues for lineage barcode research.

In conclusion, the evolving landscape of scLT represents a dynamic frontier in cellular biology. The integration with innovative technologies, such as single-cell multiomics and spatial transcriptomics, has not only addressed existing limitations but has also unveiled unprecedented opportunities for unraveling intricate clonal dynamics at both the molecular and spatial levels. As we continue to refine methodologies and expand our toolkit, the path forward holds great promise for deeper insights into cellular differentiation, tissue development, and disease progression, ultimately shaping the future of scLT research. Recognizing current gaps in knowledge, future investigations should focus on advancing computational tools, developing novel barcode libraries, and integrating multiomics approaches and spatial transcriptomics. These endeavors will undoubtedly propel our understanding further, opening new frontiers in the exploration of cellular heterogeneity and dynamic processes.
